# Tuning aromaticity of cyclocarbons by heteroatom doping: C_12_S and C_12_N

**DOI:** 10.1093/nsr/nwaf472

**Published:** 2025-10-31

**Authors:** Luye Sun, Yuan Guo, Ihor Sahalianov, Zheng Zhou, Wei Zheng, Wenzhi Xiang, Yumeng Guo, Yuanhao Feng, Rashid Valiev, Artem Kuklin, Hans Ågren, Glib V Baryshnikov, Wei Xu

**Affiliations:** Interdisciplinary Materials Research Center, School of Materials Science and Engineering, Tongji University, Shanghai 201804, China; Interdisciplinary Materials Research Center, School of Materials Science and Engineering, Tongji University, Shanghai 201804, China; Laboratory of Organic Electronics, Department of Science and Technology, Linköping University, Norrköping 60174, Sweden; Wallenberg Initiative Materials Science for Sustainability, Department of Science and Technology, Linköping University, Norrköping 60174, Sweden; Interdisciplinary Materials Research Center, School of Materials Science and Engineering, Tongji University, Shanghai 201804, China; Interdisciplinary Materials Research Center, School of Materials Science and Engineering, Tongji University, Shanghai 201804, China; Interdisciplinary Materials Research Center, School of Materials Science and Engineering, Tongji University, Shanghai 201804, China; Interdisciplinary Materials Research Center, School of Materials Science and Engineering, Tongji University, Shanghai 201804, China; Interdisciplinary Materials Research Center, School of Materials Science and Engineering, Tongji University, Shanghai 201804, China; Department of Chemistry, University of Helsinki, Helsinki FI-00014, Finland; Department of Physics and Astronomy, Uppsala University, Uppsala SE-75120, Sweden; Department of Physics and Astronomy, Uppsala University, Uppsala SE-75120, Sweden; Faculty of Chemistry, Wroclaw University of Science and Technology, Wroclaw PL-50370, Poland; Laboratory of Organic Electronics, Department of Science and Technology, Linköping University, Norrköping 60174, Sweden; Wallenberg Initiative Materials Science for Sustainability, Department of Science and Technology, Linköping University, Norrköping 60174, Sweden; Interdisciplinary Materials Research Center, School of Materials Science and Engineering, Tongji University, Shanghai 201804, China

**Keywords:** molecular carbon allotrope, cyclocarbon, doubly aromatic, heteroatom doping, on-surface synthesis, atom manipulation

## Abstract

Cyclo[*n*]carbons (C*_n_*) have sparked substantial interest among experimentalists and theoreticians owing to their elusive geometric structures and unique aromaticity. Composed of two-coordinated sp-hybridized carbon atoms, C*_n_* thus forms two perpendicular conjugated π-electron systems, i.e. out-of-plane and in-plane. Till now, on-surface generated cyclocarbons are either doubly aromatic or doubly anti-aromatic, as the number of electrons within out-of-plane and in-plane π systems was equal. Doping with heteroatoms allows one to create two π systems with different numbers of electrons, and to tune the aromaticity. Herein, we successfully generated two heteroatom-doped cyclocarbons, C_12_S and C_12_N, and characterized their chemical and electronic structures. Calculations show that C_12_S exhibits an out-of-plane (14 *e*) aromatic and in-plane (12 *e*) anti-aromatic character, resulting in a total non-aromaticity. For C_12_N, the out-of-plane (14 *e*) aromatic and in-plane (13 *e*) non-aromatic characters lead to total aromaticity. Doping with heteroatoms may open up the field of aromaticity engineering within cyclocarbons.

## Teaser

Two heteroatom-doped cyclocarbons, the totally non-aromatic C12S and totally aromatic C12N, have been synthesized and characterized on the surface using scanning probe microscopy and theoretical calculations, demonstrating the tunability of cyclocarbon aromaticity.

## INTRODUCTION

**Figure 1.**
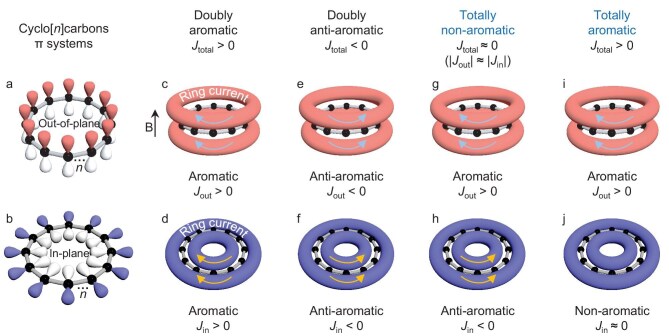
Two perpendicular π systems of cyclocarbons and their aromaticity. (a and b) Out-of-plane and in-plane π systems of cyclocarbons. (c and d) Doubly aromatic cyclocarbons with magnetically induced current *J*_total_ > 0 (*J*_out_ > 0, *J*_in_ >0). (e and f) Doubly anti-aromatic cyclocarbons with *J*_total_ < 0 (*J*_out_ < 0, *J*_in_ < 0). (g and h) Totally non-aromatic cyclocarbons with *J*_total_ ≈ 0 (e.g. *J*_out_ > 0, *J*_in_ < 0, and |*J*_out_| ≈ |*J*_in_|). (i and j) Totally aromatic cyclocarbons with *J*_total_ > 0 (e.g. *J*_out_ > 0, *J*_in_ ≈0). The external magnetic field B is perpendicular to the ring plane and points upward.

Cyclo[*n*]carbons (C*_n_*), a family of molecular carbon allotropes, have attracted significant attention owing to their elusive geometric structures and unique aromaticity [1–17]. C*_n_* possesses two perpendicular conjugated π-electron systems that are formed by the alternating triple and single bonds (or consecutive double bonds): one is in-plane and the other perpendicular to the molecular plane (i.e. out-of-plane) (Fig. 1a and b). As a natural consequence of their origin from sp-hybridized carbons, all such compounds made to date have had the same number of electrons in the two π systems. Thus, they were either doubly aromatic or doubly anti-aromatic. For example, C_6_ [18], C_10_ [19], C_14_ [19], C_18_ [20,21], and C_26_ [22] are all doubly aromatic (Fig. 1c and d); C_12_ [23], C_13_ [22], C_16_ [24], and C_20_ [23] are all doubly anti-aromatic (Fig. 1e and f).

Doping with heteroatoms opens the possibility of breaking this parity, because heteroatoms (i.e. S, N) have a lone pair in either the in-plane or out-of-plane system, but not both. Thus, exchanging a carbon atom or adding a heteroatom allows one to create two π systems with different numbers of electrons. Furthermore, it allows one to create systems with even numbers of electrons but odd numbers of atoms, or vice versa. Our calculations predict that S or N heteroatom doping into cyclocarbons (e.g. doubly anti-aromatic C_12_) could significantly influence its geometric and electronic structures, and tune its aromaticity. Herein it is shown that C_12_S exhibits out-of-plane aromatic and in-plane anti-aromatic characters, resulting in a total non-aromaticity (Fig. 1g and h), which has not been reported for any cyclocarbons so far. For C_12_N, the out-of-plane aromatic and in-plane non-aromatic characters lead to a total aromaticity (Fig. 1i and j), thus achieving a reversed aromaticity compared to C_12_. It is therefore of utmost interest to experimentally generate such heteroatom-doped cyclocarbons.

**Figure 2.**
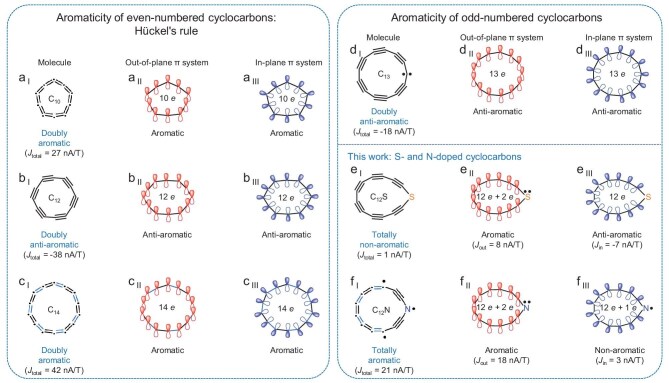
Aromaticity calculations of even- and odd-numbered cyclocarbons. (a _I_ to a _III_, b _I_ to b _III_, and c _I_ to c _III_) Aromaticity calculations of C_10_, C_12_, C_14_. (d _I_ to d _III_) Aromaticity calculations of C_13_. (e _I_ to e _III_ and f _I_ to f _III_) Aromaticity calculations of C_12_S and C_12_N.

The concept of aromaticity was introduced by Kekulé in 1865 [25], and nowadays, magnetic criteria of aromaticity are most widely used for molecular systems [16,22,26]. For the magnetically induced ring current *J*, diatropic current (*J* > 0) corresponds to aromatic character, while paratropic current (*J* < 0) corresponds to anti-aromatic one. Mathematically, we can represent the total magnetically induced current (*J*_total_) of the system as a sum of the out-of-plane and in-plane currents (i.e. *J*_out_ + *J*_in_) (cf. Fig. 1). As for even-numbered cyclocarbons shown in Fig. 2a to c (i.e. C_10_, C_12_, C_14_), the out-of-plane and in-plane π systems both contain 10 *e* (14 *e*) for aromatic C_10_ (C_14_) (Fig. 2a and c), with *J*_total_ = 27 nA/T (*J*_total_ = 42 nA/T) [11]. For anti-aromatic C_12_ (Fig. 2b), both out-of-plane and in-plane π systems contain 12 *e*, with *J*_total_ = −38 nA/T. As odd-numbered cyclocarbons, e.g. C_13_ (Fig. 2d), the out-of-plane and in-plane π systems both contain 13 *e*, with *J*_total_ = −18 nA/T, thus, C_13_ can be assigned as a doubly anti-aromatic cyclocarbon [22].

Herein, we focus on two heteroatom-doped cyclocarbons, i.e. S- and N-doped cyclocarbons. For C_12_S (Fig. 2e), calculations indicate out-of-plane (12 *e* (C) + 2 *e* (S), *J*_out_ = 8 nA/T) aromatic and in-plane (12 *e* (C), *J*_in_ = −7 nA/T) anti-aromatic character, resulting in a total non-aromaticity (*J*_total_ = 1 nA/T). For C_12_N (Fig. 2f), the out-of-plane (12 *e* (C) + 2 *e* (N), *J*_out_ = 18 nA/T) aromatic and in-plane (12 *e* (C) + 1 *e* (N), *J*_in_ = 3 nA/T) non-aromatic characters lead to a total aromaticity (*J*_total_ = 21 nA/T).

## RESULTS AND DISCUSSION

**Figure 3.**
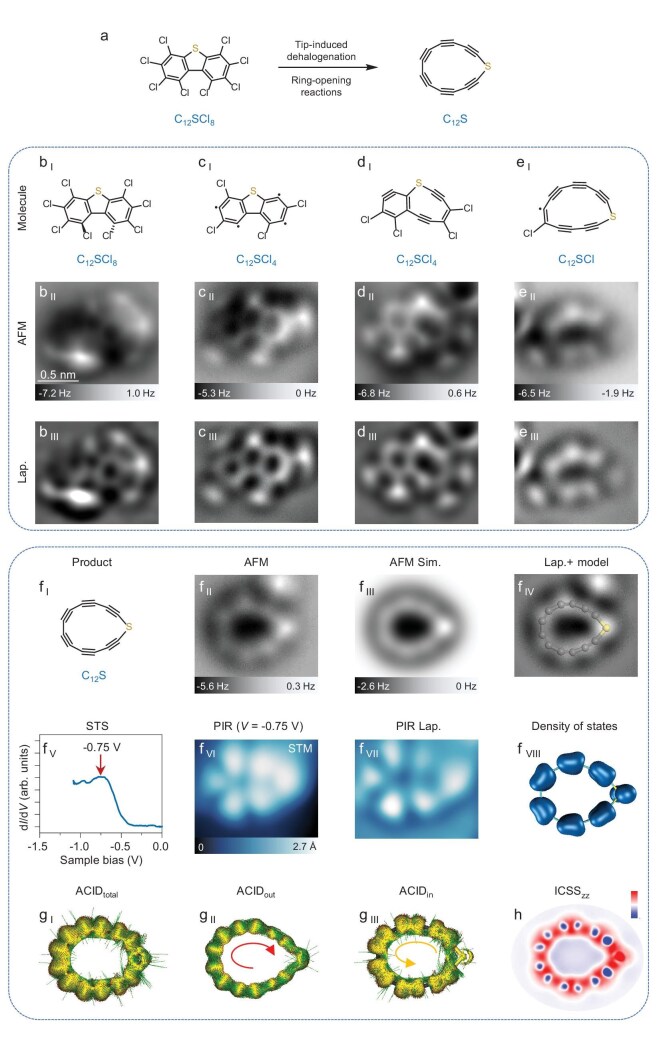
On-surface synthesis of C_12_S and its aromaticity calculations. (a) Reaction scheme for the formation of C_12_S by dehalogenation and ring-opening reactions. (b _I_ to b _III_, c _I_ to c _III_, d _I_ to d _III_, and e _I_ to e _III_) Molecular structures, AFM and Laplace-filtered AFM images of precursor and typical intermediates. (f _I_ to f _IV_) Molecular structure, AFM image, AFM simulation, Laplace-filtered AFM image with a superimposed model of product (C_12_S). (f _V_) Scanning tunneling spectroscopy (STS) of C_12_S acquired with a CO-terminated tip. The differential conductance (d*I*/d*V*) signal acquired on the C_12_S shows a peak that can be attributed to the positive ion resonance (PIR) state. (f _VI_) STM image at PIR (*V* = −0.75 V, *I* = 2 pA). (f _VII_) Laplace-filtered STM image. (f _VIII_) Superposition of the densities of the nearly energy degenerated highest occupied molecular orbitals (HOMOs). (g _I_ to g _III_) Total, out-of-plane and in-plane ACID plots for C_12_S. (h) ICSS_zz_ plot for C_12_S. Color bar: from −60 to 60. AFM tip offsets Δ*z*:  +0.3 Å, +0.2 Å, 0 Å, −0.4 Å, −1.0 Å for b _II_ to f _II_. Reference set points of Δ*z*: *I* = 5 pA, *V* = 0.3 V for b _II_, *I* = 4 pA, *V* = 0.3 V for c _II_ to e _II_, *I* = 0.5 pA, *V* = 0.3 V for f _II_. The scale bar in (b _II_) applies to all experimental images. The external magnetic field is perpendicular to the ring plane and points upward.

**Figure 4.**
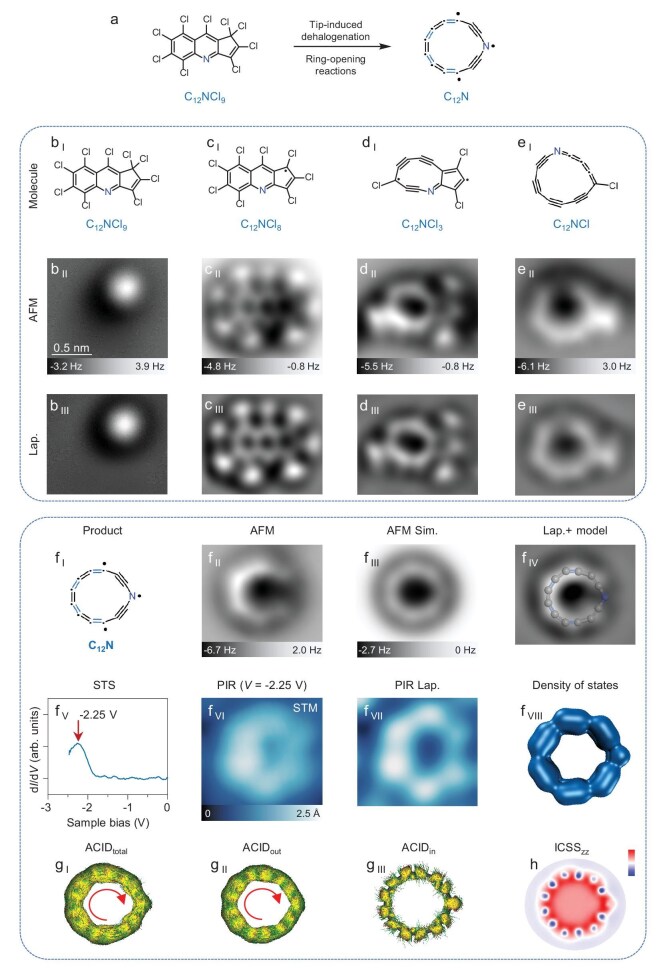
On-surface synthesis of C_12_N and its aromaticity calculations. (a) Reaction scheme for the formation of C_12_N by dehalogenation and ring-opening reactions. (b _I_ to b _III_, c _I_ to c _III_, d _I_ to d _III_, and e _I_ to e _III_) Molecular structures, AFM and Laplace-filtered AFM images of precursor and typical intermediates. (f _I_ to f _IV_) Molecular structure, AFM image, AFM simulation, Laplace-filtered AFM image with a superimposed model of product (C_12_N). (f _V_) Scanning tunneling spectroscopy (STS) of C_12_N acquired with a CO-terminated tip. The d*I*/d*V* signal acquired on the C_12_N shows a peak that can be attributed to the PIR state. (f _VI_) STM image at PIR (*V* = −2.25 V, *I* = 2 pA). (f _VII_) Laplace-filtered STM image. (f _VIII_) Superposition of the densities of the nearly energy degenerated highest occupied molecular orbitals (HOMOs). (g _I_ to g _III_) Total, out-of-plane and in-plane ACID plots for C_12_N. (h) ICSS_zz_ plot for C_12_N. Color bar: from −100 to 100. AFM tip offsets Δ*z*:  0 Å, −1.4 Å, −1.4 Å, −1.3 Å, −1.5 Å for b _II_ to f _II_. Reference set points of Δ*z*: *I* = 0.5 pA, *V* = 0.3 V for b _II_ to f _II_. The scale bar in (b _II_) applies to all experimental images. The external magnetic field is perpendicular to the ring plane and points upward.

Experimentally, we designed and synthesized two fully halogenated molecules (perchlorodibenzo[*b, d*]thiophene, C_12_SCl_8_, and perchloro-1H-cyclopenta[*b*]quinoline, C_12_NCl_9_) as the precursors for generating heteroatom-doped cyclocarbons, C_12_S and C_12_N, respectively. Through scanning tunneling microscopy (STM) tip-induced dehalogenation [27,28] and accompanied ring-opening reactions, C_12_S (cf. Fig. 3a) and C_12_N (cf. Fig. 4a) were successfully generated on the surface.

To generate C_12_S, C_12_SCl_8_ molecules were introduced on the cold sample held at ∼6 K. All molecules were studied on the 1 monolayer (ML) NaCl/Au(111) surface at 4.7 K. Atomic force microscopy (AFM) images (Fig. 3b  _II_, b _III_) acquired with a CO-terminated tip revealed the skeleton of the precursor (Fig. 3b  _I_). To trigger dehalogenation reactions, the tip was initially positioned on a single C_12_SCl_8_ molecule, and retracted by 4–6 Å from a set point (typically *I* = 5 pA, *V* = 0.3 V), after that, ∼4–4.5 V pulses were applied on the molecule with currents on the order of a few pA. Normally, several Cl atoms were removed, leading to the formation of typical intermediates shown in Fig. 3c and d and Fig. S2. In some intermediates the first-step retro-Bergman ring-opening reaction [19,23,29–31] has occurred, leading to the formation of a 9-membered ring containing a S atom (Fig. 3d and Fig. S2b). Subsequent voltage pulses can induce further dehalogenation and accompanied second-step retro-Bergman ring-opening reaction, leading to the formation of intermediates, e.g. C_12_SCl (Fig. 3e). AFM imaging shows a 13-membered ring with five carbon-carbon triple bonds and one Cl atom attached. Further voltage pulses could induce complete dehalogenation of intermediates (e.g. Fig. 3e), resulting in the formation of the final product C_12_S (cf. the optimized structure shown in Fig. 3f  _I_, Figs S3 and S4a). AFM images (Fig. 3f  _II_, f _IV_) reveal six characteristic bright features corresponding to carbon-carbon triple bonds and a pronounced contrast at the S atom site, in consistence with AFM simulation (Fig. 3f  _III_). In the close tip-sample distance (Fig. S5), bright thin lines appear between triple bonds (obviously different from the cumulenic line features) [18,19], which should originate from the tip-tilting effect [32].

Moreover, we have successfully measured the differential conductance as a function of voltage, d*I*/d*V*, of C_12_S (Fig. 3f  _V_). The d*I*/d*V* spectrum acquired over the C_12_S ring shows a prominent peak at ∼−0.75 V. STM images (Fig. 3f  _VI_, f _VII_) obtained at this bias voltage correspond to the PIR state, showing characteristic lobes similar to the ones of C_20_ [23]. This state could result from the superposition of the densities of the nearly energy degenerated highest occupied molecular orbitals (HOMOs) (Fig. 3f  _VIII_ and Fig. S6). It is considered that the peak at the PIR dominantly relates to the out-of-plane orbitals [22]. In addition, due to the energy broadening of the ionic resonances on NaCl (∼0.3 V) [33], nearly degenerated orbitals could not be resolved as separate peaks in the d*I*/d*V* spectrum.

C_12_NCl_9_ molecules [34] (Fig. 4b  _I_ to b _III_) were introduced onto the surface to generate C_12_N. Similarly, applied voltage pulses (∼4–4.5 V) can remove one or more Cl atoms, leading to the formation of typical intermediates (e.g. Fig. 4c–e). Not only the 6–6–5 skeleton, but more importantly the larger 10- and 13-membered rings of intermediates were revealed by AFM images, indicating the occurrence of first-step and second-step ring-opening reactions. Subsequent voltage pulses could induce complete dehalogenation of intermediates (e.g. Fig. 4e), resulting in the formation of the final product C_12_N (cf. the optimized structure shown in Fig. 4f  _I_, Figs S4b and S7). For C_12_N, the N atom site in AFM images (Fig. 4f  _II_, f _IV_) has a weaker contrast compared to carbon-carbon bonds [35]. AFM images at different tip heights (Fig. 4f  _II_ and Fig. S8) show two characteristic bright features (assigned to triple bonds) near to the N site and uniform line features (assigned to the cumulenic moiety) within C_12_N, in accordance with the AFM simulation (Fig. 4f  _III_).

Moreover, the d*I*/d*V* spectrum (Fig. 4f  _V_) acquired over the C_12_N ring shows a prominent peak at ∼−2.25 V. STM images (Fig. 4f  _VI_, f _VII_) obtained at this bias voltage correspond to the PIR state, showing a nearly delocalized state (i.e. no obvious lobes in comparison with C_12_S) (Fig. 4f  _VIII_ and Fig. S9). It is still challenging to measure the negative ion resonance (NIR) of C_12_S and C_12_N due to the high mobility of the ring.

Calculations for anisotropy of the induced current density (ACID) [36] were conducted to visualize the magnetically induced current of C_12_S (Fig. 3g and Fig. S10). ACID_out_ (Fig. 3g  _II_) and ACID_in_ (Fig. 3g  _III_) plots indicate the presence of a diatropic current (indicated by red clockwise arrow) within the out-of-plane π system and paratropic current (indicated by yellow anti-clockwise arrow) within the in-plane π system, leading to a very small diatropic current in ACID_total_ (Fig. 3g  _I_). It is found that an in-plane paratropic current is formed by avoiding the kink at the S atom, because the S atom could not provide the lone electron pair into the conjugation circuit inside the C_12_S ring, thus only in-plane 12 electrons by 12 carbon atoms are involved in the paratropic current. Iso-chemical shielding surface (ICSS_zz_) [37] plots (Fig. 3h and Fig. S11) further confirm the presence of diatropic out-of-plane and paratropic in-plane currents.

For C_12_N, ACID_out_ (Fig. 4g  _II_) and ACID_in_ (Fig. 4g  _III_) plots indicate the presence of a large diatropic current (indicated by red clockwise arrow) within the out-of-plane π system and very small diatropic current within the in-plane π system, leading to a diatropic current (indicated by red clockwise arrow) in ACID_total_ (Fig. 4g  _I_ and Fig. S12). Compared with C_12_S, the N atom provides only one electron into the in-plane conjugation circuit of the C_12_N ring, resulting in a 13π-electron very weak aromatic system, while the out-of-plane 14π-electron aromatic system provides the dominant diatropic contribution to the total current. Moreover, the ICSS_zz_ plots only show a diatropic contribution (Fig. 4h and Fig. S13). The results of the aromaticity calculations of C_12_S and C_12_N are in consistence with the geometries and electronic state revealed by AFM and STM imaging (Figs 3f and 4f). Specifically, compared with the non-aromatic C_12_S, aromatic C_12_N exhibits reduced bond-length alternations and more delocalized PIR states.

## CONCLUSION

In conclusion, we have successfully generated two heteroatom S- and N-doped cyclocarbons, i.e. C_12_S and C_12_N, via the on-surface synthesis method. The doped heteroatoms result in different numbers of electrons within the out-of-plane and in-plane conjugated π systems, and tune the aromaticity. Such a strategy of introducing heteroatoms may open up the field of aromaticity engineering within cyclocarbons.

## Supplementary Material

nwaf472_Supplemental_File
