# Supplementary material for: Tuning aromaticity of cyclocarbons by heteroatom doping: C12S and C12N
Source: Natl Sci Rev. 2025 Oct 31;13(7):nwaf472. doi: 10.1093/nsr/nwaf472 (PMC13056706; doi:10.1093/nsr/nwaf472)
Supplement: nwaf472_Supplemental_File [file nwaf472_supplemental_file.pdf]

Tuning aromaticity of cyclocarbons by heteroatom doping: $C_{12}S$ and $C_{12}N$

Luye Sun[1,7], Yuan Guo[1,7], Ihor Sahalianov[2,3,7], Zheng Zhou[1,7], Wei Zheng[1,7], Wenzhi Xiang[1], Yumeng Guo[1], Yuanhao Feng[1], Rashid Valiev[4], Artem Kuklin[5], Hans Ågren[5,6], Glib V. Baryshnikov[2,3,]*, Wei Xu[1,]*

[1]Interdisciplinary Materials Research Center, School of Materials Science and Engineering, Tongji University, Shanghai 201804, People's Republic of China.

[2]Laboratory of Organic Electronics, Department of Science and Technology, Linköping University, 60174 Norrköping, Sweden.

[3]Wallenberg Initiative Materials Science for Sustainability, Department of Science and Technology, Linköping University, 60174 Norrköping, Sweden.

[4]Department of Chemistry, University of Helsinki, FI-00014 Helsinki, Finland.

[5]Department of Physics and Astronomy, Uppsala University, Box 516, SE-75120 Uppsala, Sweden.

[6]Faculty of Chemistry, Wroclaw University of Science and Technology, Wyb. Wyspianskiego 27, PL-50370 Wroclaw, Poland.

[7]These authors contributed equally: Luye Sun, Yuan Guo, Ihor Sahalianov, Zheng Zhou, Wei Zheng

*Corresponding authors. Email: glib.baryshnikov@liu.se, xuwei@tongji.edu.cn

The PDF file includes:

Materials and Methods
Tables S1 and S2
Figs. S1 to S18
Relaxed geometries
References

Materials and Methods

<u>Solution synthesis of perchlorodibenzo[*b*,*d*]thiophene ($C_{12}SCl_8$)</u>

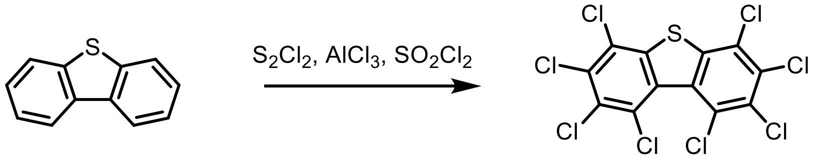

Preparation of $C_{12}SCl_8$

To a suspension of $AlCl_3$ (850 mg, 6.38 mmol) in SO_2Cl_2 (75 mL) was added a solution of dibenzothiophene (74 mg, 0.4 mmol) in S_2Cl_2 (2.0 mL, 25 mmol) and SO_2Cl_2 (30 mL) over 30 min via a syringe pump. In the meantime, the solution was stirred at 65 ℃. After the completion of the addition, the solution was stirred at 65 ℃ for 24 h. After cooling to room temperature, CH_2Cl_2 (30 mL) was added. The mixture was slowly added into water at 0 ℃, followed by quenching with an aqueous saturated Na_2CO_3 solution. The layers were separated, and the aqueous phase was extracted with CH_2Cl_2. The organic phases were combined, washed with brine, dried and filtered. Solvent removal and washing with CH_2Cl_2 afforded decachlorofluorene as a white powder (90 mg, 0.2 mmol, 50 %). No signals from the product were observed.

<u>Synthesis of perchloro-1H-cyclopenta[*b*]quinoline ($C_{12}NCl_9$)</u>

Chemicals and materials

PCl_5 (99.0%) and acridine (98.0%) were purchased from Adamas and used as received.

Preparation of $C_{12}NCl_9$

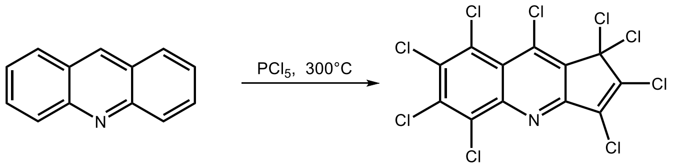

A custom-made autoclave with Hastelloy alloy (150 mL) was charged with acridine (0.932 g, 0.0052 mol) and PCl_5 (40 g, 0.192 mol), which was heated rapidly to 300 °C (1.5 hours) and then maintained at this temp for a further 16 hours. The autoclave was allowed to cool to room temperature and vented to release the HCl formed during the reaction before the vessel was opened. The product was then hydrolyzed by the slow addition of ice. When this was complete, the chlorinated product was filtered off and dried.

The mixture was purified by dynamic sublimation at 130 ℃ for 1 h to obtain a yellow solid, and the yellow solid was sublimated in a glass ampule (O.D. 1 cm, L. 15 cm) at 130–135 ℃ for 5 days, giving colorless block crystals suitable for single crystal X-ray

diffraction in good yield (Yield: 90%).

Structure solution and refinement details

Data collection of $C_{12}NCl_9$ was performed on a Bruker VENTURE system equipped with a PHOTON III C14 detector, a Cu-target iμs 3.0 microfocus X-ray source (λ = 1.54178 Å), and a graphite monochromator. The data were collected at T = 150(2) K (Oxford Cryosystems CRYOSTREAM 1000). Data reduction and integration were performed with the Bruker APEX5 software package SAINT (version 8.40B). Data were corrected for absorption effects using the empirical methods as implemented in SADABS (version 2016/2). The structures were solved by SHELXT (version 2018/2)[1] and refined by full-matrix least-squares procedures using the Bruker SHELXTL (version 2019/2)[2] software package through the OLEX2 graphical interface[3]. All non-hydrogen atoms, including those in disordered parts, were refined anisotropically. Hydrogen atoms were included in idealized positions for structure factor calculations with $U_{iso}(H) = 1.2\ U_{eq}(C)$. Crystallographic data and details of the data collection and structure refinement are listed in Table S2. The crystal structure is shown in Fig. S1.

Experimental details for STM and AFM measurements

STM and AFM measurements were carried out in a commercial (Createc) low-temperature system operated at 4.7 K with a base pressure better than 1×10^{-10} mbar. The single crystalline Au(111) surface was cleaned by several sputtering and annealing cycles. The NaCl films were obtained by thermally evaporating NaCl crystals onto a clean Au(111) surface at room temperature, all experiments were performed on the one monolayer (ML) NaCl surface. The perchlorodibenzo[*b*,*d*]thiophene ($C_{12}SCl_8$) and perchloro-1H-cyclopenta[*b*]quinoline ($C_{12}NCl_9$) precursor molecules were synthesized as detailed in the supporting information. The $C_{12}SCl_8$ and $C_{12}NCl_9$ molecules were separately deposited on a cold NaCl/Au(111) surface by thermal sublimation from a molecular evaporator. CO molecules for tip modification[4] were dosed onto the cold sample via a leak valve. We used qPlus sensors[5] with a resonance frequency f_0 = 29.49 kHz, quality factor $Q \approx$ 45,000 and a spring constant $k \approx 1800$ N/m operating in frequency-modulation mode[6]. The bias voltage V was applied to the sample with respect to the tip. AFM images were acquired in constant-height mode at V = 0 V and an oscillation amplitude of A = 1 Å. The tip-height offsets Δz for constant-height AFM images are defined as the offset in tip-sample distance relative to the STM set point at the NaCl surface. The positive (negative) values of Δz correspond to the tip-sample distance increased (decreased) with respect to a STM set point.

Theoretical methods

The optimization of electronic ground state molecular structure was performed using the complete active space self-consistent field (CASSCF) and complete active space perturbation theory at the second order (XMC-CASPT2)[7] in the BAGEL software[8]. The 8 electrons in 8 molecular orbitals (MOs) and the SVP basis set were used. The magnetically induced current strengths were calculated using the Ampère–Maxwell law[9]. This method requires the nuclear magnetic resonance (NMR) shielding constants for atoms of molecules and placement of fifty dummy atoms over the center of the molecule. NMR shielding constants were calculated using the CASSCF(8x8), restricted active space self-consistent field (RASSCF)[10] based on CASSSCF and CASPT2 geometry in the DALTON software[11]. In the case of RASSCF, the additional 4 MOs in RAS1, 4MOs in RAS3 with a maximum of two electronic excitations were considered. In the case of the WB97XD/6-311++g(d,p) method the NMR shielding constants were calculated using GAUSSIAN 16[12]. The magnetically induced current density was simulated using the GIMIC[13] software and PARAVIEW[14].

We also fully optimized the geometry of $C_{12}S$ and $C_{12}N$ with the 6-311++g(d,p)[15] basis set followed by a check to verify the absence of negative frequencies. Simulations were carried out with B3LYP[16,17], BHandHLYP[18], BMK[19], M062X[20], PBE[21], and ωB97XD[22] exchange-correlation functionals. Electrostatic potential surfaces were extracted with cubegen utility and plotted by using Chemcraft software[23]. Iso-chemical shielding surfaces ($ICSS_{zz}$) were generated using the Multiwfn 3.8 software[24]. Anisotropy of induced current density (ACID) plots were generated by considering both σ- and π-orbitals and calculated by a method, developed by Herges et al[25].

Density functional theory (DFT) calculations with plane-wave basis sets were carried out using the D3 corrected[26] Perdew-Burke-Ernzerhof (PBE) exchange functional[21] as implemented in the Vienna Ab-initio Simulation Package (VASP)[27,28]. We employed the projector augmented wave (PAW) method[29] with a plane-wave basis set cutoff energy of 500 eV. The calculations were carried out in the gamma point of the first Brillouin zone (BZ). Convergence tolerances for forces and electronic minimizations were set to 10^{-2} eV/Å and 10^{-5} eV, respectively. To prevent any spurious interaction in the z-direction, the vacuum region was set to at least ~20 Å. The Visualization for Electronic and Structural Analysis (VESTA) software[30] was used to plot atomic structures and isosurfaces, and the "VASPKIT" code was used for postprocessing the results[31].

The AFM simulations were conducted by the PP-AFM code provided by Hapala *et al*[32]. The detailed parameters are listed below. The lateral spring constant for CO-tip was 0.2 N/m, and a quadrupole-like charge distribution at the tip apex was used to simulate the CO tip with q = -0.1 e. In addition, e is the elementary charge and refers

to |*e*|, and *q* is the magnitude of quadrupole charge at the tip apex. The amplitude was set as 1 Å.

Calculations of the magnetically induced ring current J_{total}

Our results predict the total magnetically-induced current strength J_{total} = 1 nA/T and 21 nA/T for $C_{12}S$ and $C_{12}N$, respectively. In GIMIC simulations, we used the procedure to estimate J_{in} and J_{out} contributions to the total current J_{total}.

1) By setting up the grid size across the bond where we calculate the current. The resulting J_{in} was found to be −7 nA/T and 3 nA/T for $C_{12}S$ and $C_{12}N$, respectively, when we used grid size only inside the molecule. However, J_{out} of 8 nA/T and 18 nA/T for $C_{12}S$ and $C_{12}N$ was calculated using the grid size only outside the molecule. This exactly corresponds to the total currents J_{total} = 1 nA/T and 21 nA/T for $C_{12}S$ and $C_{12}N$.

2) By calculating the orbital contributions. This option was recently realized in GIMIC code for closed-shell systems only[33]. Taking $C_{12}S$ as an example, by using a classification of orbitals in four groups (core, σ, π-in, π-out) analogously to ACID calculations, we have obtained the total current of 1.56 nA/T. From Table S1 one can see that core orbitals provide a considerable diatropic contribution to the J_{total}, which is compensated by the overall paratropic current contribution from σ-skeleton orbitals. The summation over core and σ-skeleton orbitals results in a weak diatropic current of 3.24 nA/T. The π-in orbitals expectedly provided a strong paratopic current of -10.41 nA/T. One should note that HOMO orbital 44 (J_{44} = -22.26 nA/T) is a predominant contributor to J_{in}. The π-out orbitals provide a diatropic current of 8.73 nA/T, which partially compensates paratropic π-in contribution resulting in a total π-in + π-out current of -1.68 nA/T. Thus, if consider only the π-in and π-out electrons, we obtained the conclusion about net current close to zero but weakly paratropic. However, with accounting of core+σ diatropic contribution the net magnetically induced current remains close to zero but becomes weakly diatropic.

Additional calculations on the aromaticity of $C_{12}S$ and $C_{12}N$.

1) ACID simulations.

All the ACID simulations were performed for the series of optimized geometries at different exchange-correlation functionals (PBE0, B3LYP, ωB97XD, BhandLYP, BMK) and 6-311++g(d,p) basis set. These results are presented in supplementary materials and are qualitatively the same for all functionals. Following our experience with related cyclocarbons, we analyzed in more detail the aromaticity of $C_{12}S$ and $C_{12}N$ on ωB97XD-optimized geometries. Typically, the continuous set of gauge transformations (CGST) approach was used at the first stage to calculate the magnetic shielding tensors. After that, the ACID software[25] was employed to generate the ACID plots. The isosurface value was chosen to be 0.05, the length of current vectors was set to 3, and the vector of the magnetic field was oriented perpendicularly to the cyclocarbon plane. Partial orbital contributions to ACID plots were simulated for the selected orbitals based on orbital analysis, provided before.

2) ICSSzz simulations

Iso-chemical shielding surfaces (zz component) were generated in Multiwfn 3.8 software [25] by using the methodology, developed by Liu et al.[34] Magnetic shielding tensor was calculated for chosen grid points, revealing the extent of shielding effects caused by delocalized electrons. Visualization was performed with three 2D maps in different planes (XY, XZ, YZ). We employed gauge independent atomic orbital (GIAL) approach for simulations of NMR shielding tensors based on optimized geometries at the chosen functional (PBE0, B3LYP, wB97XD, BhandLYP, BMK) and 6-311++g(d,p) basis set. We used a low-quality grid option (130910 points[25]) to generate a template Gaussian input file. After running all of them sequentially, we generated an ICSSzz cube file, which was used for visualization in Multiwfn.

3) HOMA

As was calculated and mentioned in the manuscript, $C_{12}S$ presents near total non-aromaticity (J_{total} = 1 nA/T), and $C_{12}N$ presents total aromaticity (J_{total} = 21 nA/T). However, cyclocarbons represent a special case of aromatic systems sustaining strong bond alternations, and HOMA criterion which is based on structural deviations of target systems from ideal benzene reference cannot be correctly applied to describe their aromaticity. Indeed, HOMA indexes[35] in classic parametrization (α = 94.09 for C-S, α = 93.52 for C-N, α = 257.7 for C-C) are even negative: -2.52 for $C_{12}S$ and -1.81 for $C_{12}N$ (were extracted with Multiwfn). This indicates that more accurate methods are needed to investigate the aromaticity of cyclocarbons, while HOMA is a rather primitive method that doesn't capture the electron structure of the cyclocarbons.

4) FLU simulations

FLU[36-38] and FLU-π are only available for closed-shell systems in Multiwfn. A lower FLU value indicates greater delocalization and thus higher aromaticity, with an ideal aromatic system like benzene having a FLU index of 0. Because the reference parameter for C-S is absent in Multiwfn, we extracted only FLU-π for $C_{12}S$. After performing NBO analysis at wB97XD/6-311++g(d,p) level of theory. We used the produced .fchk file as input to Multiwfn. At first, π-out-orbitals were identified as 32,

34, 35, 38, 39, 42, 43. After that, we used functions 15-7 and obtained an FLU-π-out value of 0.278. At the same time, FLU-π-in value for orbitals 31, 33, 36, 37, 40, 41, 44 was extracted as 0.297, which is similar but slightly larger than FLU-π-out. This indicates weaker aromaticity within the π-in system. If we consider the fact that the S atom does not take part in π-in delocalization, the FLU-π-in index extracted only for the twelve carbon atoms on the same geometry excluding S from the sequence was equal to value 0.481, which indicates much weaker aromaticity within π-in system with respect to π-out one. Thus, we conclude that simulated FLU-π indexes for $C_{12}S$ agree with the magnetic criteria of aromaticity (ACID, ICSSzz, GIMIC).

5) MCBO simulations

The most severe drawback of the MCBO is its high basis set dependency. In particular, if diffuse functions are presented, the MCBO result may be misleading or completely meaningless. That is the reason for choosing a Multi-centre index (MCI) on a natural atomic orbital basis, implemented in Multiwfn. Similarly to FLU, MCI is not realized for open-shell systems in Multiwfn. As for $C_{12}S$, the multicentre bond order was calculated as 0.00057, and the normalized multicentre bond order 0.56320, which indicates it is less aromatic than benzene (the multicentre bond order: 0.08709 and the normalized multicenter bond order: 0.66578).

Table S1. Diatropic, paratropic, and total currents for core, σ, π-in, and π-out orbitals for $C_{12}S$. Units: nA/T for all values.

	Diatropic	Paratropic	Total
core	11.22	-1.1	10.11
σ	31.25	-38.1	-6.87
π-in	20.5	-30.92	-10.41
π-out	20.91	-12.19	8.73

Table S2. Crystal data and structure refinement parameters for $C_{12}NCl_9$.

Compound	$C_{12}NCl_9$
CCDC Number	2387696
Empirical formula	$C_{12}NCl_9$
Formula weight	477.18
Temperature (K)	150(2)
Wavelength (Å)	1.54178
Crystal system	Orthorhombic
Space group	*Pnma*
a (Å)	38.1882(18)
b (Å)	6.7886(3)
c (Å)	19.7046(6)
α (°)	90
β (°)	90
γ (°)	90
V (Å^3)	3156.6(3)
Z	8
ρ_{calcd} (g cm^{-3})	2.008
μ (mm^{-1})	14.550
F(000)	1856
Crystal size (mm)	0.05×0.06×0.20
θ range for data collection (°)	3.810-71.995
Reflections collected	14823
Independent reflections	3318
	[R_{int} = 0.0308]
Transmission factors (min/max)	0.3411/0.7536
Data/restraints/params.	3318/0/259
$R1$,[a] $wR2$[b] ($I > 2\sigma(I)$)	0.0326, 0.0838
$R1$,[a] $wR2$[b] (all data)	0.0328, 0.0839
Quality-of-fit[c]	1.150

$R_{int} = \Sigma|F_o^2 - \langle F_o^2 \rangle| / \Sigma|F_o^2|$

[a] $R1 = \Sigma||F_o|-|F_c|| / \Sigma|F_o|$. [b] $wR2 = [\Sigma[w(F_o^2-F_c^2)^2] / \Sigma[w(F_o^2)^2]]$.

[c] Quality-of-fit = $[\Sigma[w(F_o^2-F_c^2)^2] / (N_{obs}-N_{params})]^{½}$, based on all data.

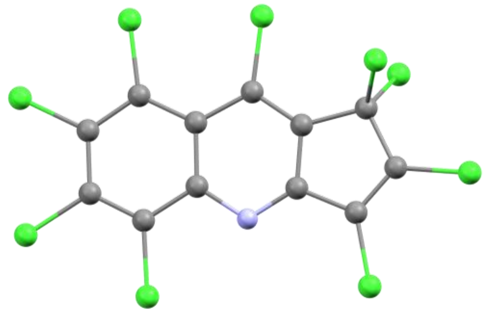

Fig. S1. Molecular structure of $C_{12}NCl_9$ obtained from SC-XRD experiment in ball and stick model.

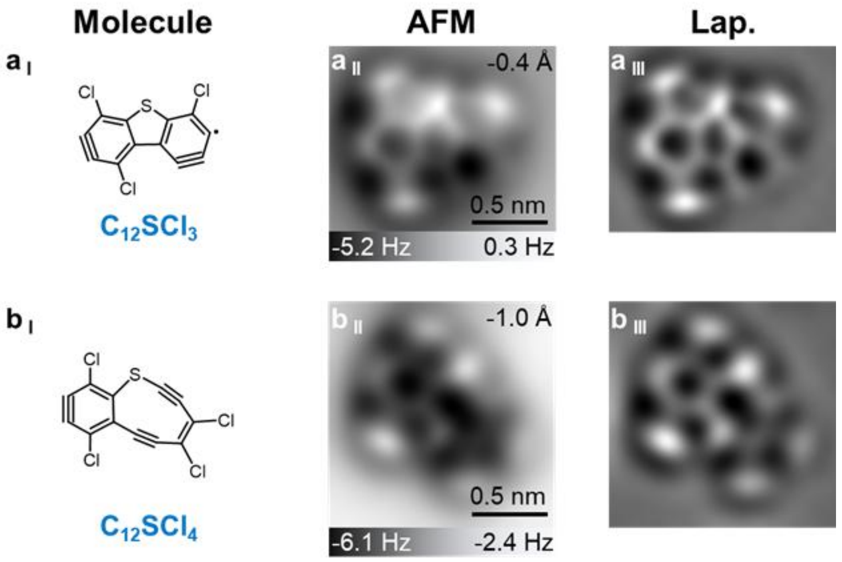

Fig. S2. Schematic molecular structures, AFM images and Laplace-filtered AFM images of other observed intermediates. (**a** $_{\mathrm{I}}$ to **a** $_{\mathrm{III}}$) $C_{12}SCl_3$, (**b** $_{\mathrm{I}}$ to **b** $_{\mathrm{III}}$) $C_{12}SCl_4$. Reference set point of Δz: I = 4 pA, V = 0.3 V.

a

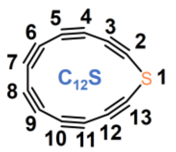

C-C	Bond length(Å)
1-2	1.692
2-3	1.215
3-4	1.359
4-5	1.219
5-6	1.356
6-7	1.222
7-8	1.357
8-9	1.222
9-10	1.356
10-11	1.219
11-12	1.359
12-13	1.215
13-1	1.692

C-C-C	Bond angle (°)
1-2-3	164.368
2-3-4	166.187
3-4-5	156.343
4-5-6	156.660
5-6-7	148.830
6-7-8	148.268
7-8-9	148.418
8-9-10	148.750
9-10-11	156.677
10-11-12	156.349
11-12-13	166.160
12-13-1	164.395
13-1-2	98.596

b

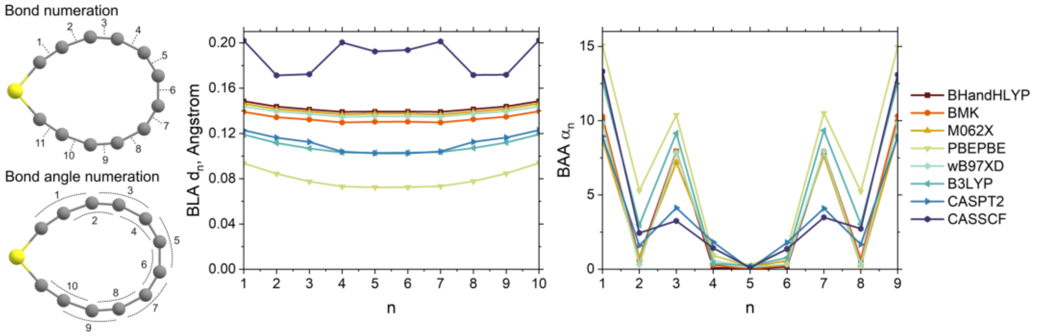

Fig. S3. Structural characterization of $C_{12}S$. (**a**) The bond lengths and bond angles in $C_{12}S$. Calculations were conducted at the ωB97XD/6-311++g(d,p) level. (**b**) Bond length alternation (BLA) and bond angle alternation (BAA) descriptors calculated at different levels of theory.

Optimization of $C_{12}S$ structure with different levels of theory always leads to the structure with clear bond length alternation (BLA d_n) between adjacent CC bonds l_{n+1} and l_n ($d_n = |l_{n+1} - l_n|$). There is a clear trend that the BLA parameter increases with the rise of HF exchange within the functional starting from 0.08-0.10 Å for the pure PBEPBE functional until approximately 0.15 Å for BHandHLYP functional (Fig. S3b). CASSCF(8x8) level of theory overestimates the BLA in $C_{12}S$ at the level 0.17-0.20 Å due to the lack of accounting of dynamic correlation effects. At the same time XMC-CASPT2 simulations provide a the reasonable BLA in line with DFT results and most closely relate to the optimization with the B3LYP functional. In contrast to BLA, the bond angle alternation BAA ($\alpha_n = |L_{n+1} - L_n|$) only slightly varies among different functionals (7.5 º-10 º), while CASSCF and XMC-CASPT2 methods result in much smaller BAA deviation at the level of 3-4 °(Fig. S3b).

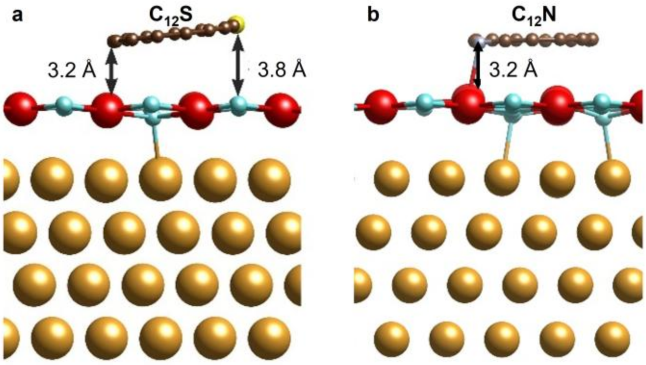

Fig. S4. Optimized equilibrium geometry of $C_{12}S$ (a) and $C_{12}N$ (b) adsorbed on NaCl/Au(111).

To accurately model the real experimental conditions, it is essential to use periodic boundary conditions and take into account substrate effects. Therefore, we performed calculations on the adsorption of $C_{12}S$ adsorbed on NaCl/Au(111) using the periodic boundary PBC approximation. The initial model of the interface consisted of a five-layer Au (111) surface with deposited NaCl (001) monolayer which was matched and created using the Virtual NanoLab (VNL) tool[39,40] to minimize mutual structural stress. We determined that the unit cell is large enough (23.93 × 19.94 Å) to simulate $C_{12}S$ adsorption on the NaCl/Au(111) while avoiding spurious interactions between the molecules, maintaining an effective separation distance of 14.5 Å between neighboring molecules. The simulated $C_{12}S$/NaCl/Au(111) heterostructure is shown in Fig. S4a. In contrast to cyclo[18]carbon, which reveals cumulene type when the PBE functional with 0% HFE was used[41], the present $C_{12}S$ molecule demonstrates bond length alternation (BLA) with short and long bond lengths of 1.256 and 1.329 Å, respectively. Therefore, we believe that the PBE functional can qualitatively describe the adsorption properties of $C_{12}S$. We found that the adsorption energy is ~ -0.03 eV and varies depending on the position. The global energy minimum was found when the S atom is ~3.8 Å above Cl, while two carbon atoms on the opposite side of the molecule are ~3.2 Å above Na. Therefore, the molecule is not parallel to the surface.

Similarly to $C_{12}S$, we performed calculations on the adsorption of $C_{12}N$ on NaCl/Au(111) using the periodic boundary (PBC) approximation. The simulated $C_{12}N$/NaCl/Au(111) heterostructure is shown in Fig. S4b. In contrast to the $C_{12}S$ molecule which demonstrates bond length alternation (BLA) with short and long bond lengths of 1.256 and 1.329 Å, the present $C_{12}N$ radical reveals a much smaller BLA varying between 1.278 and 1.309 Å at the PBE level of theory. We found that the ground state adsorption energy is very low (~ -0.36 eV) and varies depending on the position. The global energy minimum was found when the N atom is ~3.2 Å above Na. It is also found that the molecule is located parallel to the surface.

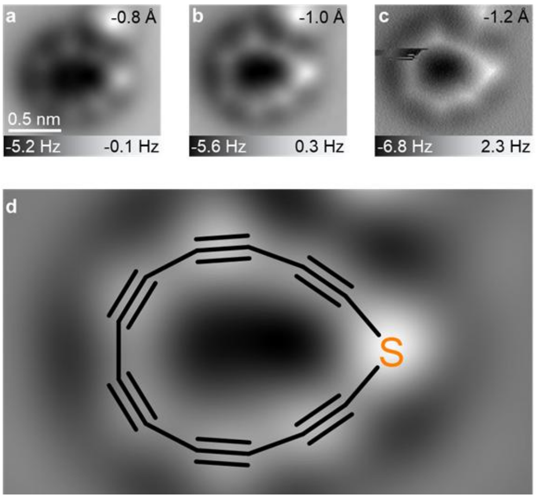

Fig. S5. AFM images of $C_{12}S$ at different tip heights. AFM images (**a** to **c**) of $C_{12}S$ at different tip heights (Δz) acquired with a CO-terminated tip. (**d**) Laplace-filtered AFM image of $C_{12}S$ superimposed with molecular model. Reference set point of Δz: I = 0.5 pA, V = 0.3 V.

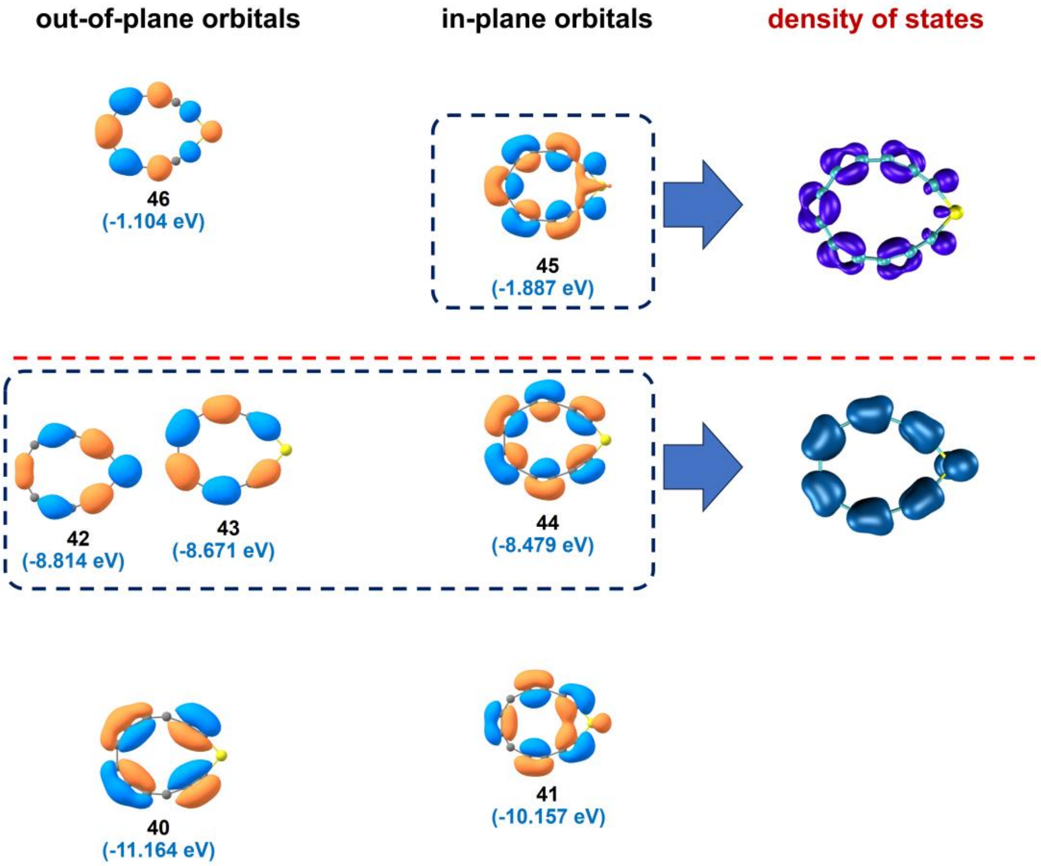

Fig. S6. DFT calculations of frontier orbitals of $C_{12}S$ in the gas phase. Superpositions of orbital densities of the nearly energetically degenerated orbitals are also shown.

a

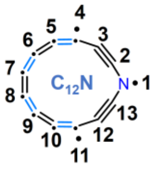

C-C	Bond length(Å)
1-2	1.273
2-3	1.245
3-4	1.316
4-5	1.257
5-6	1.305
6-7	1.262
7-8	1.300
8-9	1.262
9-10	1.305
10-11	1.257
11-12	1.316
12-13	1.245
13-1	1.273

C-C-C	Bond angle (°)
1-2-3	174.288
2-3-4	142.082
3-4-5	165.449
4-5-6	141.123
5-6-7	158.640
6-7-8	148.570
7-8-9	148.496
8-9-10	158.692
9-10-11	141.081
10-11-12	165.472
11-12-13	142.082
12-13-1	174.293
13-1-2	119.731

b

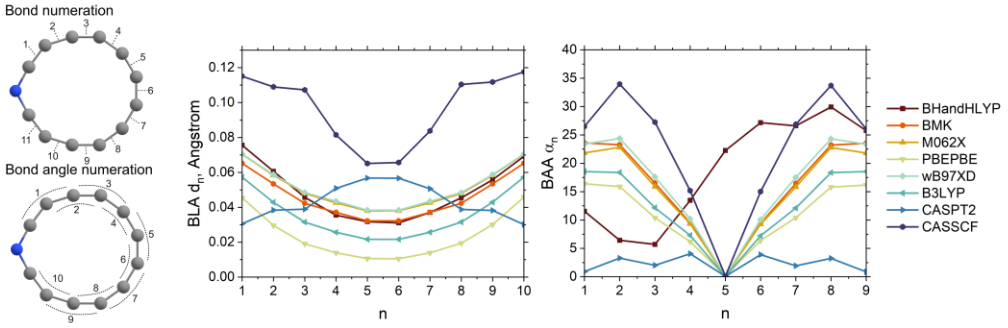

Fig. S7. Structural characterization of $C_{12}N$. (**a**) The bond lengths and bond angles in $C_{12}N$. Calculations were conducted at the ωB97XD/6-311++g(d,p) level. (**b**) Bond length alternation (BLA) and bond angle alternation (BAA) descriptors calculated at different levels of theory.

We have considered the structure of $C_{12}N$ in its ground state by using DFT and *ab initio* calculations. We have found that the $C_{12}N$ radical sustains the round-shaped form with a slight kink at the CNC centre. This is the sequence of delocalization of the unpaired electron over the whole $C_{12}N$ ring but not localizing on the N atom. The trends in BLA are closely similar between all the DFT methods which predict it in the range 0.01-0.08 Å. CASSCF(8x8) optimized structure shows a similar BLA trend to DFT with a more pronounced magnitude (Fig. S7b), while the CASPT2 method predicts the other trend of BLA which approximately corresponds to the inversion of bond orders in the ring (co-called bond shift). At the same time, the BAA descriptor (Fig. S7b) demonstrates basically the same trend with all employed methods, while

only the BHandHLYP functional predicts a distorted geometry of $C_{12}N$ which is probably the impact of the high percentage of HF exchange in the functional.

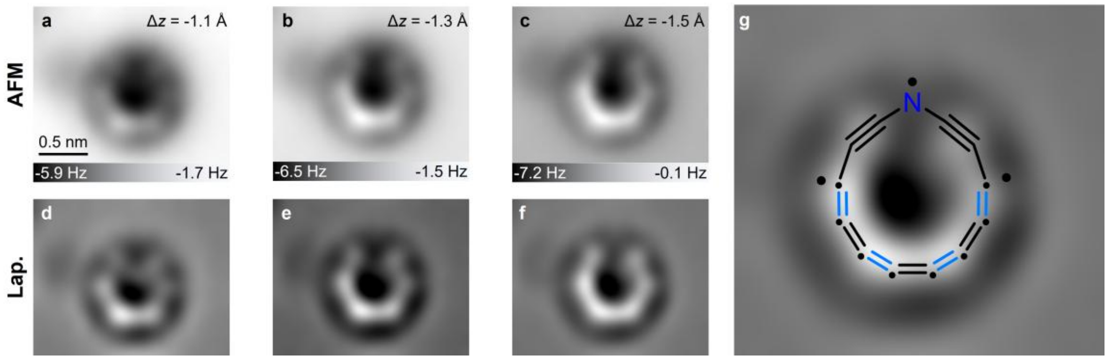

Fig. S8. AFM images of $C_{12}N$ at different tip heights. AFM images (**a** to **c**) and Laplace-filtered AFM images (**d** to **f**) of $C_{12}N$ at different tip heights (Δ*z*) acquired with a CO-terminated tip. (**g**) Laplace-filtered AFM image of $C_{12}N$ superimposed with molecular model. Reference set point of Δ*z*: *I* = 0.5 pA, *V* = 0.3 V.

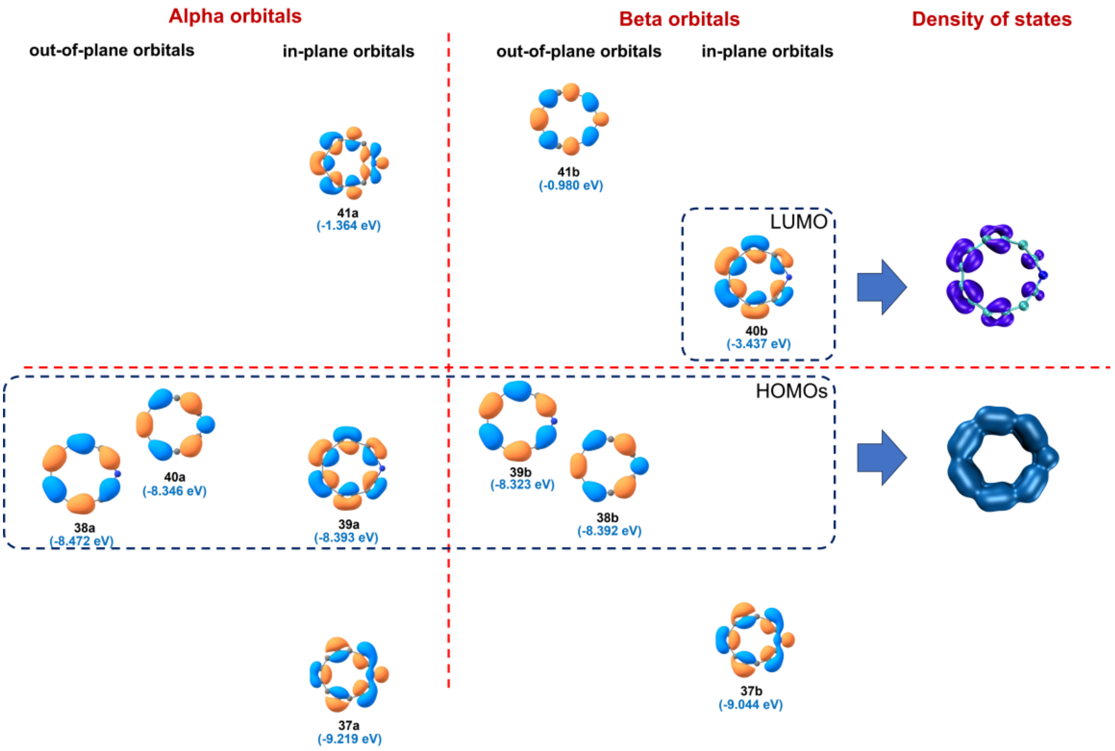

Fig. S9. DFT calculations of frontier orbitals of $C_{12}N$ in the gas phase. Superpositions of orbital densities of the nearly energetically degenerated orbitals are also shown.

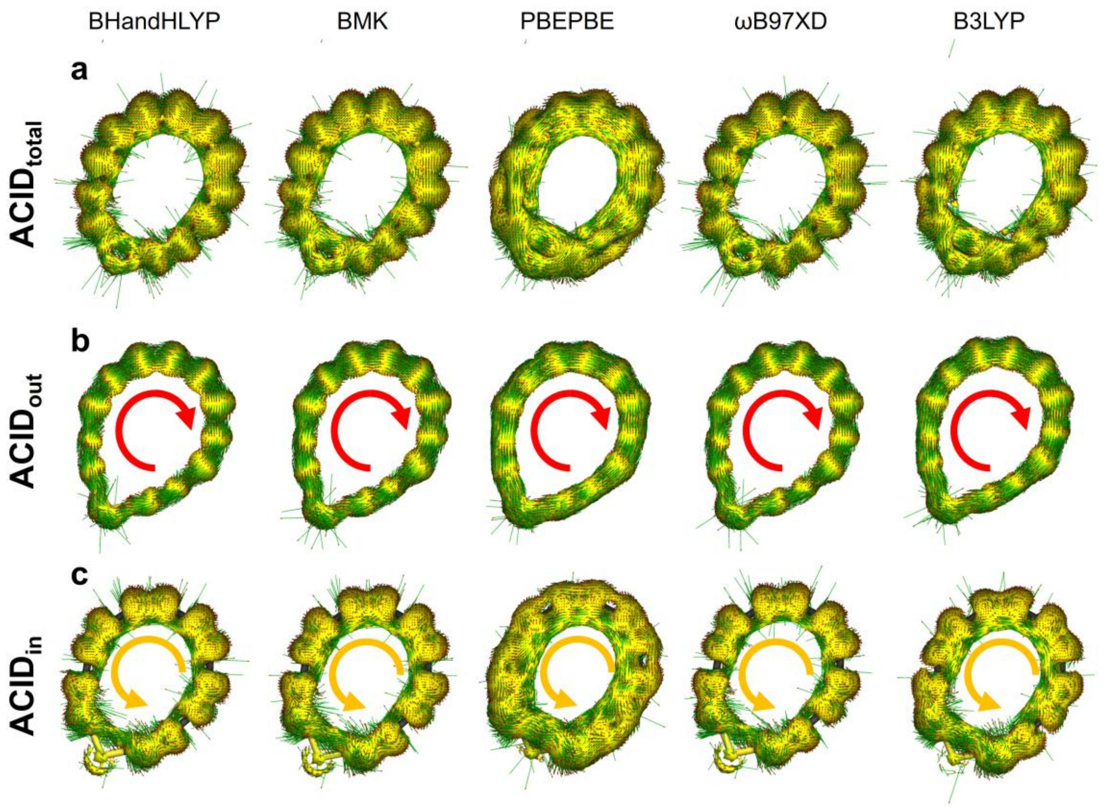

Fig. S10. ACID plots for $C_{12}S$ calculated at different levels of DFT. (**a**) $ACID_{total}$. (**b**) $ACID_{out}$. (**c**) $ACID_{in}$. The external magnetic field **B** is perpendicular to the ring plane and points upward.

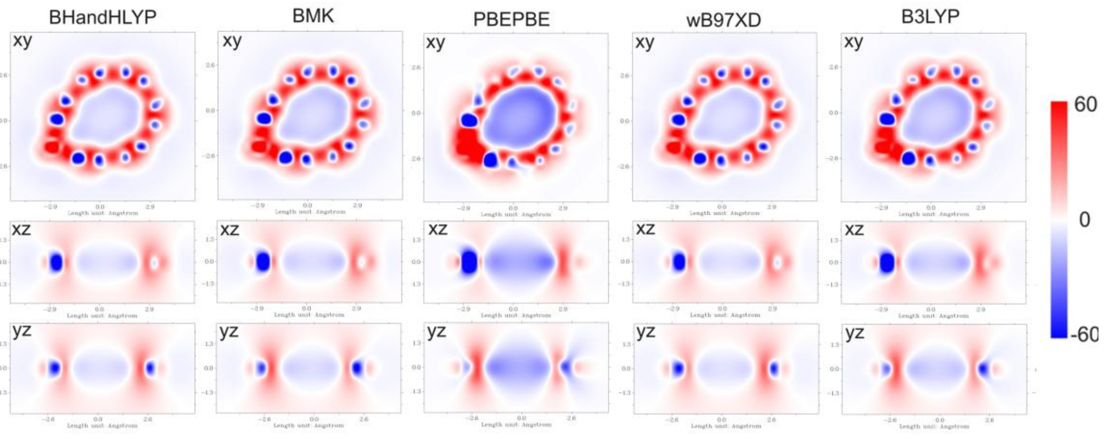

Fig. S11. 2D ICSSzz plots for $C_{12}S$ molecule calculated by different DFT methods and presented in different projections. The external magnetic field **B** is perpendicular to the ring plane and points upward.

ICSSzz plots (Fig. S11) confirms the presence of diatropic out-of-plane and paratropic in-plane induced currents–there is clear area inside the ring (xy projections) characterized by negative values of the magnetic shielding tensor (zz-component) indicating the antiaromaticity and out-of-plane area (xz and yz projections) characterized by positive values of magnetic shielding tensor (zz-component) indicating the aromaticity. It is also clearly seen from the xz and yz projections in the ICSS plots that the negative shielding area (blue colour) is limited in space only by the area inside the ring, while the positive shielding area is localized up and down with respect to the molecular plane and envelop the negative shielding area.

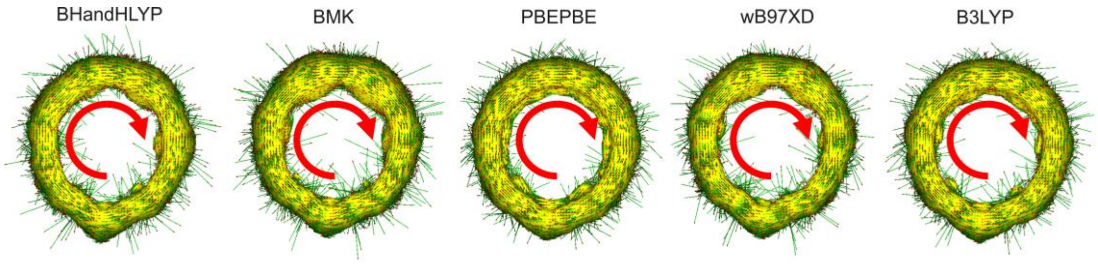

Fig. S12. Total ACID plots for $C_{12}N$ calculated at different levels of DFT. The external magnetic field **B** is perpendicular to the ring plane and points upward.

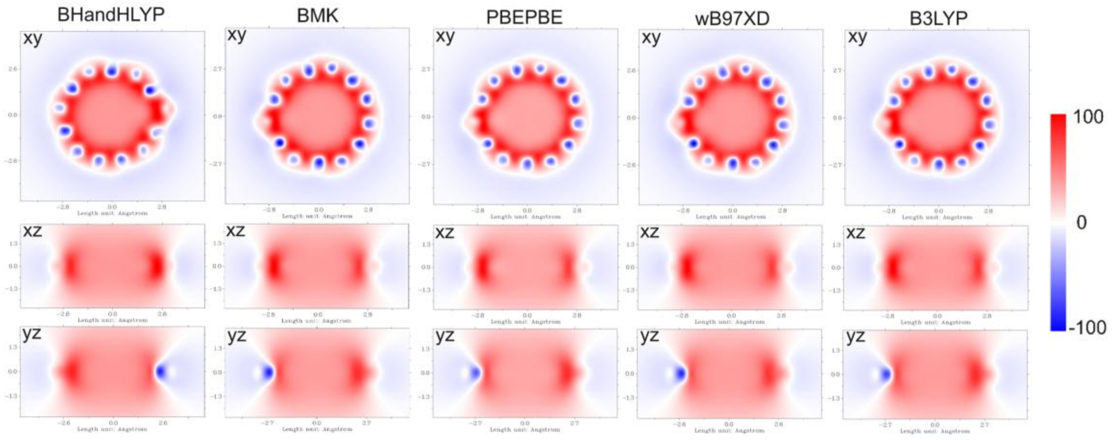

Fig. S13. 2D $ICSS_{zz}$ plots for $C_{12}N$ molecule calculated at different DFT methods and presented in different projections.

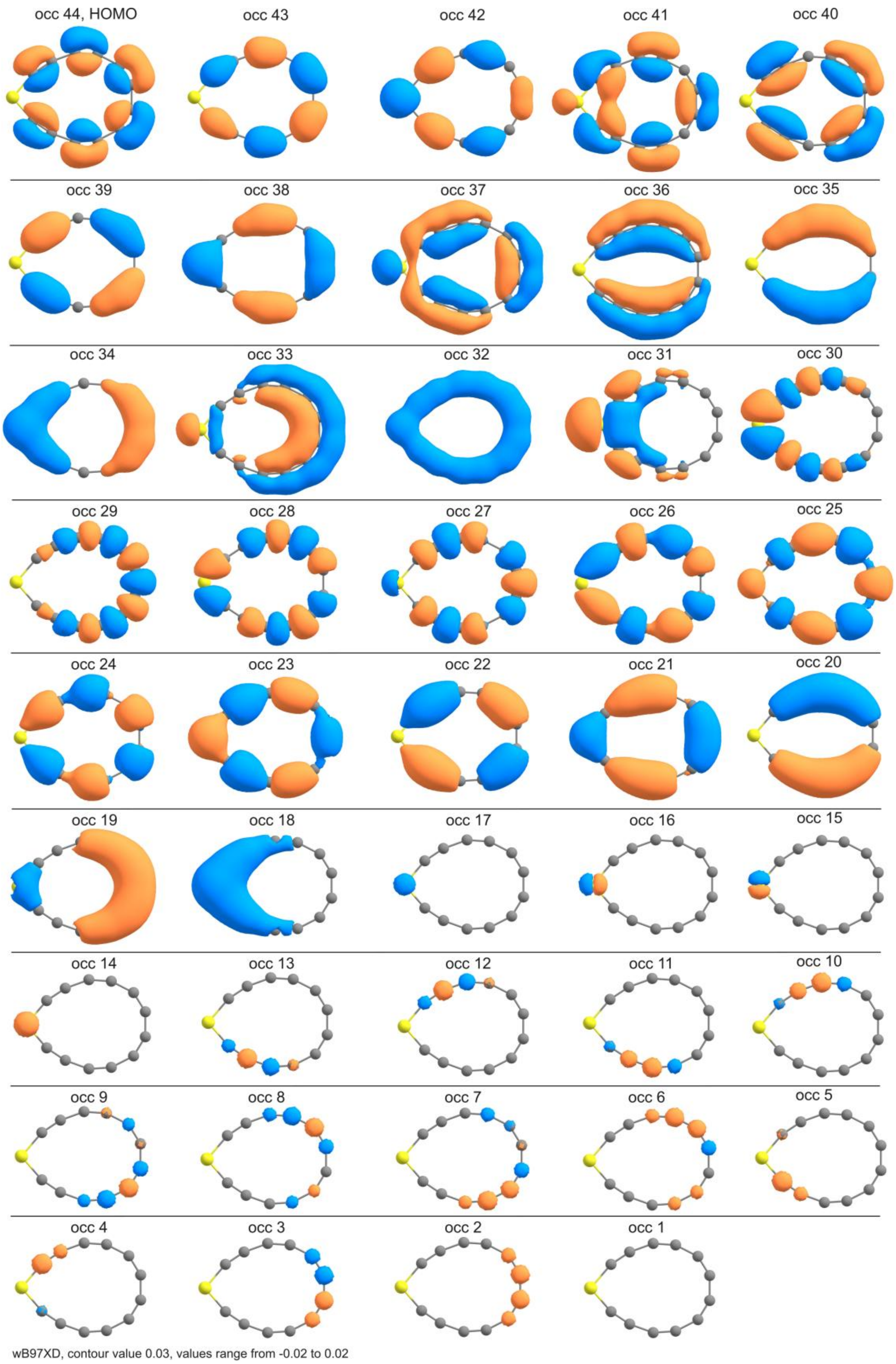

Fig. S14. Molecular orbitals in $C_{12}S$.

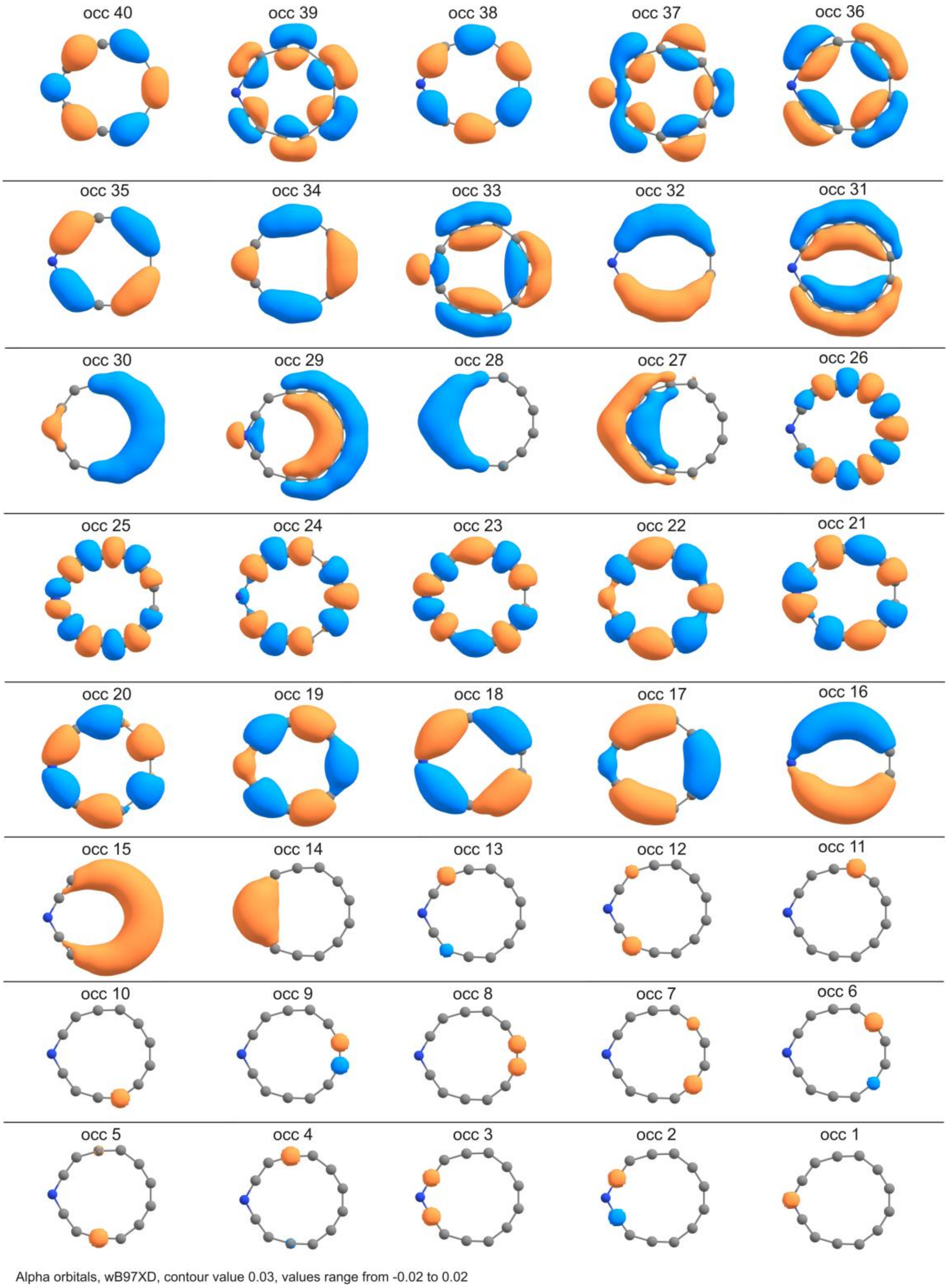

Fig. S15. Molecular orbitals in $C_{12}N$ (Alpha spin type).

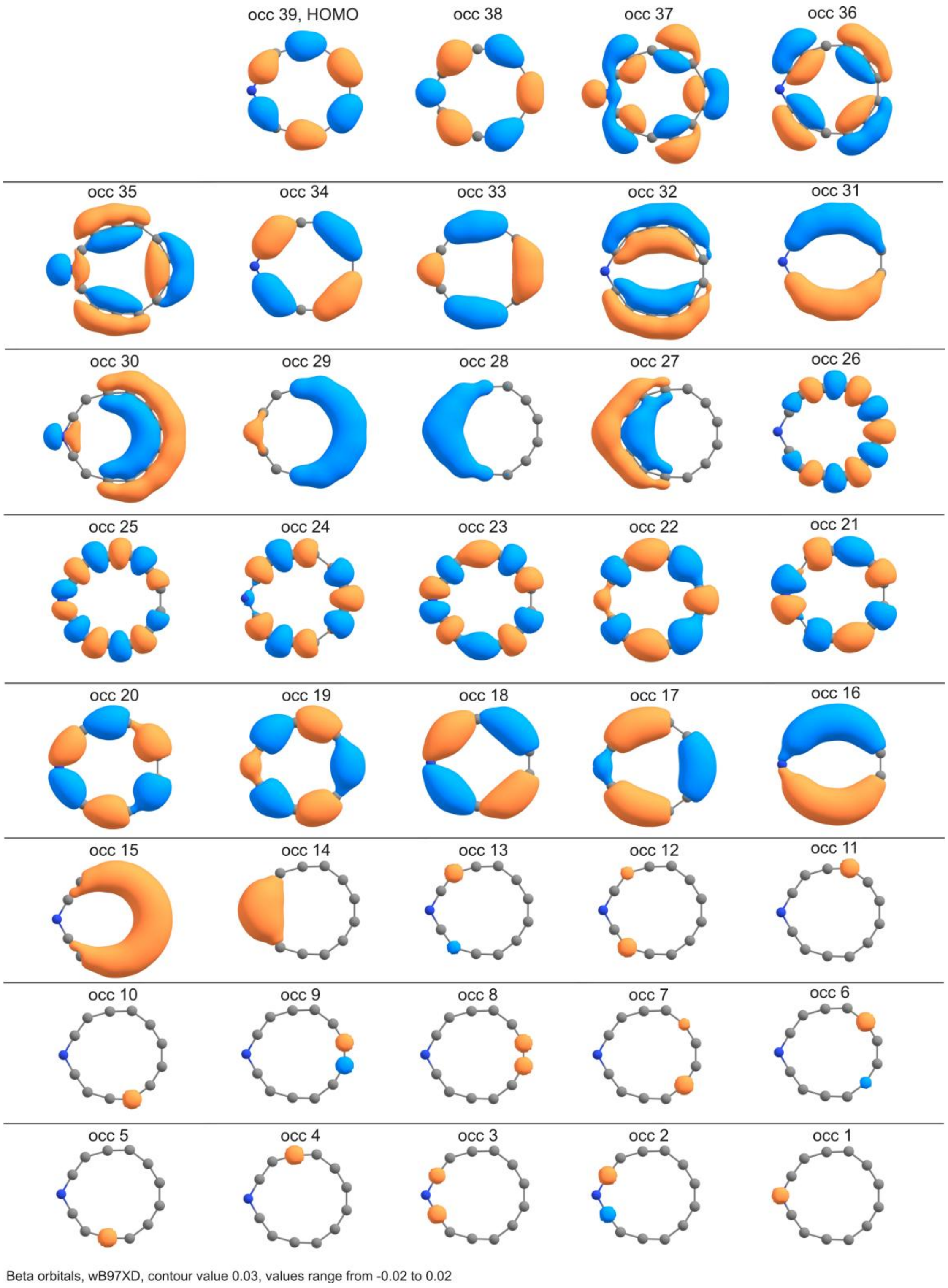

Fig. S16. Molecular orbitals in $C_{12}N$ (Beta spin type).

Orbital calculations of $C_{12}S$ and $C_{12}N$. The total number of electrons in $C_{12}S$ is 88. As follows from orbital analysis, the lowest 17 orbitals can be classified as "core" orbitals: twelve orbitals (24 electrons) correspond to $1s^2$ core electrons of twelve carbon atoms and five remaining orbitals (10 electrons) correspond to $1s^22s^22p^6$ core electrons of sulfur atom. One can see from Fig. S14 that the core orbitals of S are listed as 1,14,15,16,17 corresponding to $1s^2$, $2s^2$, $2p_x^2$, $2p_y^2$, $2p_z^2$ respectively. The next thirteen orbitals (numbers 18-30) correspond to a σ-bond skeleton containing 13 σ-bonds. Orbitals 31,33,36,37,40,41,44 (seven in total) are classified as in-plane π-orbitals (π-in) and the remaining orbitals 32,34,35,38,39,42,43 are out-of-plane π-orbitals (π-out). This classification agrees with the results of natural population analysis (NPA).

For $C_{12}N$ the total number of electrons is 79 and in spin-unrestricted DFT formalism, they correspond to 40α and 39β singly-occupied molecular orbitals. Similar to $C_{12}S$, the lowest 26 (13α+13β) orbitals in $C_{12}N$ correspond to core $1s^2$ electrons of 12 carbons and 1 nitrogen. From Fig. S15 and S16, one can see the strict spatial symmetry equivalence between these 13α and 13β (orbitals). The next 26 orbitals (13α+13β) correspond to a σ-bond skeleton containing 13 σ-bonds analogously to the $C_{12}S$ case. The orbitals 27α, 29α, 31α, 33α, 36α, 37α together with 27β, 30β, 32β, 35β, 36β, 37β symmetrical counterparts and standalone orbital 39α correspond to 13 π-in orbitals.

Thus, we confirm that for the case of $C_{12}S$, we have 14 π-in and 14 π-out electrons, corresponding to 7 π-in and 7 π-out doubly occupied molecular orbitals. But for the case of $C_{12}N$, we deal with 13 π-in and 14 π-out electrons, corresponding to 13 π-in and 14 π-out singly occupied molecular orbitals.

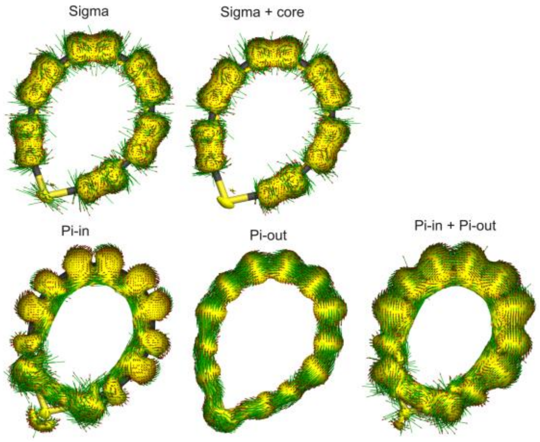

Fig. S17. ACIDs for $C_{12}S$, aggregated in five groups (σ, σ+core, π-in, π-out, π-in+π-out).

Following the orbital analysis for $C_{12}S$, we separated all 44 occupied orbitals into four different groups (core, σ, π-in, π-out) and performed ACID simulations. One can see from Fig. S17 that ACID plots for σ-skeleton orbitals and for combined

σ-skeleton+core orbitals are eventually the same and do not exhibit any total magnetically-induced currents. When we consider only seven π-in orbitals (31, 33, 36, 37, 40, 41, 44) we can see that the total paratropic (anticlockwise) current appears which only involves twelve carbon atoms avoiding the S atom kink. This corresponds to the situation when twelve π-in electrons from twelve carbon atoms form the anti-aromatic configuration, while $3s^2$ pair on sulfur does not contribute to the in-plane total current. At the same time, seven π-out orbitals form a clear fourteen-electron aromatic configuration, as follows from the ACID plot for π-out orbitals (32, 34, 35, 38, 39, 42, 43).

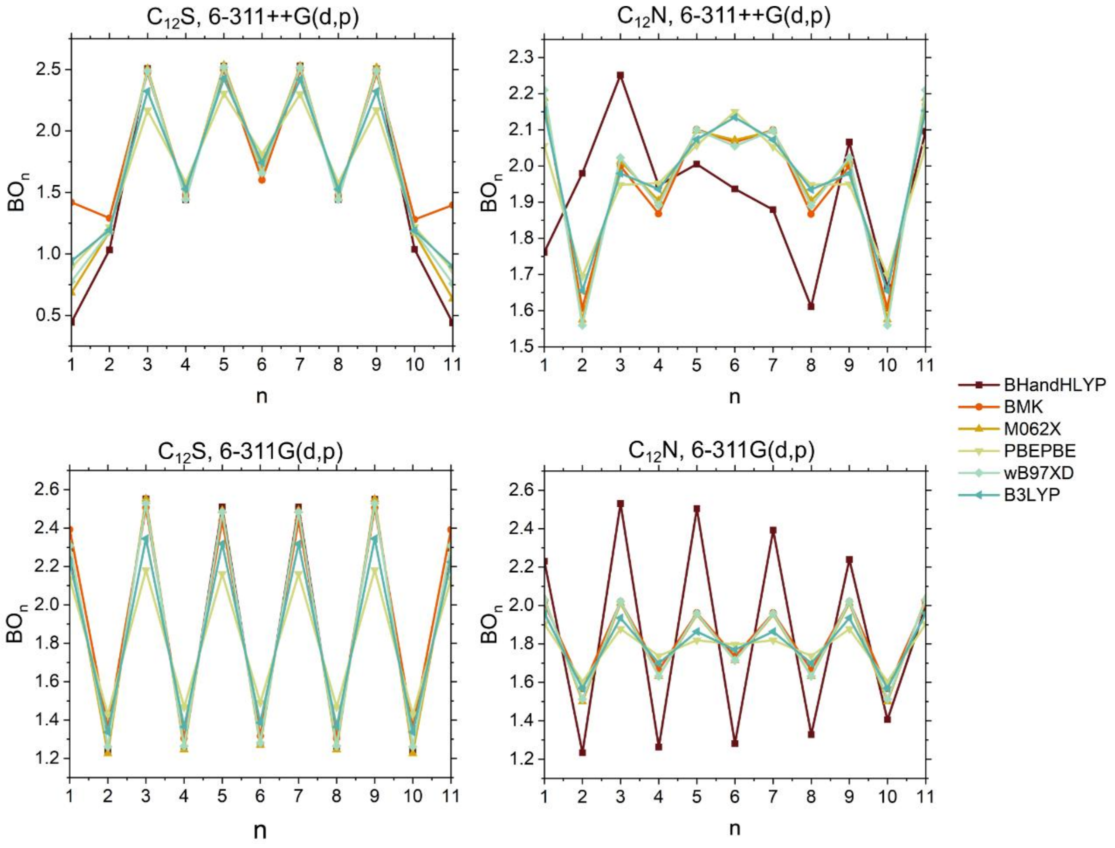

Fig. S18. Calculate Mayer bond (BO) order for $C_{12}S$ and $C_{12}N$. The calculations follow the same color-scheme as before for BLA and BAA.

<u>Relaxed structure of $C_{12}S$ using CASSCF.</u>

<u>XYZ</u>

C	0.555511306	2.475617723	0.000000000
C	1.828379836	1.892271349	0.000000000
C	-2.116331749	-1.543944745	0.000000000
C	2.575325240	0.917136959	0.000000000
C	2.741115453	-0.472947421	0.000000000
C	1.275077084	-2.512320009	0.000000000
C	2.324085115	-1.596731234	0.000000000
C	0.103440125	-2.771548075	0.000000000

C	-1.250929770	-2.415353162	0.000000000
C	-2.520317673	-0.204113971	0.000000000
C	-0.624938271	2.679246204	0.000000000
C	-2.563628425	0.992424718	0.000000000
S	-2.334376847	2.686324989	0.000000000

Relaxed structure of $C_{12}S$ using CASPT2.

XYZ

C	0.544112816	2.445615512	0.000000000
C	1.740379828	1.785441912	0.000000000
C	-2.038044450	-1.432185235	0.000000000
C	2.495218339	0.789065795	0.000000000
C	2.769084574	-0.545647512	0.000000000
C	1.342058352	-2.624357307	0.000000000
C	2.378405164	-1.742039143	0.000000000
C	0.098760862	-2.819705903	0.000000000
C	-1.175314240	-2.336745478	0.000000000
C	-2.499032780	-0.145886800	0.000000000
C	-0.663176056	2.743801199	0.000000000
C	-2.600492784	1.093500905	0.000000000
S	-2.358816104	2.772155136	0.000000000

Relaxed structure of $C_{12}S$ using ωB97XD.

XYZ

S	-2.79882100	-1.55791500	0.00000000
C	-1.21084800	-2.14215700	0.00000000
C	0.00000000	-2.23888100	0.00000000
C	1.34088600	-2.02059600	0.00000000
C	2.36439800	-1.35837900	0.00000000
C	3.11808000	-0.23070800	0.00000000
C	3.17321100	0.98967600	0.00000000
C	2.51242100	2.17468500	0.00000000
C	1.44678300	2.77203000	0.00000000
C	0.09112500	2.72524100	0.00000000
C	-1.01094200	2.20429600	0.00000000
C	-1.90309500	1.17975700	0.00000000
C	-2.45849600	0.09947400	0.00000000

Relaxed structure of $C_{12}N$ using CASSCF.

XYZ

N	2.910700901	0.306141304	0.000000000
C	2.359996969	-0.847159542	0.000000000
C	1.740344001	-1.908698593	0.000000000
C	0.496843464	-2.419333151	0.000000000

C	-0.713932112	-2.664052115	-0.000000001
C	-1.834151682	-1.924157382	0.000000000
C	-2.626661446	-0.943264707	0.000000000
C	-2.768391288	0.375285699	0.000000000
C	-2.200873004	1.500800349	0.000000001
C	-1.256402042	2.457381442	0.000000001
C	-0.022582441	2.470347313	0.000000000
C	1.300732321	2.226103719	-0.000000001
C	2.129259542	1.319582114	0.000000000

Relaxed structure of $C_{12}N$ using CASPT2.

XYZ

C	1.819387632	-1.919306808	0.000031849
C	0.691362873	-2.532977414	-0.000006062
C	-0.070394231	2.624948772	0.012903703
C	-0.628643551	-2.613219203	0.013083455
C	-1.793118589	-2.073298937	0.012971897
C	-2.815518086	0.230802906	0.021013120
C	-2.629091708	-1.033201438	0.016521002
C	-2.316934710	1.468482476	0.020851817
C	-1.357445811	2.321230848	0.026442334
C	1.186577018	2.361581551	0.011800592
C	2.583293028	-0.850766906	-0.055634749
C	2.225390494	1.558389264	-0.045440375
N	2.661540465	0.392017349	-0.029604458

Relaxed structure of $C_{12}N$ using ωB97XD.

XYZ

N	-2.87628000	-0.30252100	0.00000000
C	-2.35584800	0.85949800	0.00000000
C	-1.73636100	1.93949700	0.00000000
C	-0.51868500	2.43749600	0.00000000
C	0.72684300	2.60572900	0.00000000
C	1.84349400	1.92987200	0.00000000
C	2.61089100	0.92818500	0.00000000
C	2.74739300	-0.36484800	0.00000000
C	2.20459500	-1.50398900	0.00000000
C	1.25331500	-2.39774500	0.00000000
C	0.00000000	-2.49187300	0.00000000
C	-1.29465200	-2.25816200	0.00000000
C	-2.12532500	-1.33071900	0.00000000

$C_{12}S$ and $C_{12}N$ molecules have 26 and 27 π-in+π-out electrons respectively. Including all of them in the active space with a reasonable number of unoccupied orbitals (like CAS(26,26) or CAS(26,18)) requires huge computational resources and seems to be impossible to execute for the system of thirteen atoms. For the open-shell $C_{12}N$ case, the situation is even more complicated. In our calculations, we did not classify electrons by the shape of occupied orbitals, but we used the energy separation criterion instead. For example, the 8 electrons in 8 MOs for C12S and 7 electrons in 8 MOs for C12N are located close in their energies, while other MOs are energy separated. For example, $C_{12}N$ has the active MOs 37, 38, 39, 40, 41, 42, 43, and 44 with the energies -0.188, -0.14, -0.135, -0.124, 0.188, 0.197, 0.227, 0.235 Hartree respectively whereas the first inactive orbital 36 has the energy of -0.28 Hartree and upper 45 inactive orbital has the energy of 0.345 Hartree. Also, $C_{12}S$ has orbitals 41, 42, 43, 44, 45, 46, 47, 48 with energies -0.394, -0.339, -0.330, -0.325, 0.001, 0.02, 0.07, 0.08 Hartree respectively, whereas the first inactive lower 40 has -0.43 and upper 49 has energy of 0.11 Hartree.

References

1 Sheldrick, G. SHELXT-Integrated space-group and crystal-structure determination. *Acta Cryst. A* **71**, 3-8 (2015).

2 Sheldrick, G. Crystal structure refinement with SHELXL. *Acta Cryst. C* **71**, 3-8 (2015).

3 Dolomanov, O. V., Bourhis, L. J., Gildea, R. J., Howard, J. A. K. & Puschmann, H. OLEX2: A Complete Structure Solution, Refinement and Analysis Program. *J. Appl. Crystallogr.* **42**, 339-341 (2009).

4 Gross, L., Mohn, F., Moll, N., Liljeroth, P. & Meyer, G. The chemical structure of a molecule resolved by atomic force microscopy. *Science* **325**, 1110-1114 (2009).

5 Giessibl, F. J. High-speed force sensor for force microscopy and profilometry utilizing a quartz tuning fork. *Appl. Phys. Lett.* **73**, 3956-3958 (1998).

6 Albrecht, T. R., Grütter, P., Horne, D. & Rugar, D. Frequency modulation detection using high-*Q* cantilevers for enhanced force microscope sensitivity. *J. Appl. Phys.* **69**, 668-673 (1991).

7 Shiozaki, T. BAGEL: Brilliantly advanced general electronic-structure library. *Wiley Interdiscip. Rev. Comput. Mol. Sci.* **8**, e1331 (2018).

8 Brilliantly advanced general electronic-structure library—BAGEL Manual https://nubakery.org (accessed Jun 5, 2024).

9 Berger, R. J. F., Dimitrova, M., Nasibullin, R. T., Valiev, R. R. & Sundholm, D. Integration of global ring currents using the Ampère-Maxwell law. *Phys. Chem. Chem. Phys.* **24**, 624-628 (2022).

10 Jensen, F. *Introduction to computational chemistry*. (2007).

11 Aidas, K. *et al.* The Dalton quantum chemistry program system. *Wiley Interdiscip. Rev. Comput. Mol. Sci.* **4**, 269-284 (2014).

12 Frisch, M. J. *et al.* Gaussian 16 Rev. C.01; Gaussian, Inc.:Wallingford, CT. (2016).

13 Fliegl, H., Taubert, S., Lehtonen, O. & Sundholm, D. The gauge including magnetically induced current method. *Phys. Chem. Chem. Phys.* **13**, 20500-20518 (2011).

14 ParaView: Open-source, multi-platform data analysis and visualization application https://www.paraview.org (accessed Jun 5, 2024).

15 Hehre, W. J. Ab initio molecular-orbital theory. *Acc. Chem. Res.* **9**, 399-406 (1976).

16 Becke, A. D. Density-functional thermochemistry. III. The role of exact exchange. *J. Chem. Phys.* **98**, 5648-5652 (1993).

17 Lee, C., Yang, W. & Parr, R. G. Development of the Colle-Salvetti correlation-energy formula into a functional of the electron density. *Phys. Rev. B* **37**, 785-789 (1988).

18 Becke, A. D. A new mixing of Hartree-Fock and local density-functional theories. *J. Chem. Phys.* **98**, 1372-1377 (1993).

19 Boese, A. D. & Martin, J. M. L. Development of density functionals for thermochemical kinetics. *J. Chem. Phys.* **121**, 3405-3416 (2004).

20 Zhao, Y. & Truhlar, D. G. The M06 suite of density functionals for main group thermochemistry, thermochemical kinetics, noncovalent interactions, excited states, and transition elements: two new functionals and systematic testing of four M06-class functionals and 12 other functionals. *Theor. Chem. Acc.* **120**, 215-241 (2008).
21 Perdew, J. P., Burke, K. & Ernzerhof, M. Generalized gradient approximation made simple. *Phys. Rev. Lett.* **77**, 3865-3868 (1996).
22 Chai, J. D. & Head-Gordon, M. Long-range corrected hybrid density functionals with damped atom-atom dispersion corrections. *Phys. Chem. Chem. Phys.* **10**, 6615-6620 (2008).
23 Chemcraft - Graphical program for visualization of quantum chemistry computations https://www.chemcraftprog.com (accessed Jun 5, 2024).
24 Lu, T. & Chen, F. Multiwfn: a multifunctional wavefunction analyzer. *J. Comput. Chem.* **33**, 580-592 (2012).
25 Geuenich, D., Hess, K., Köhler, F. & Herges, R. Anisotropy of the induced current density (ACID), a general method to quantify and visualize electronic delocalization. *Chem. Rev.* **105**, 3758-3772 (2005).
26 Grimme, S. Semiempirical GGA-type density functional constructed with a long-range dispersion correction. *J. Comput. Chem.* **27**, 1787-1799 (2006).
27 Kresse, G. & Furthmüller, J. Efficient iterative schemes for *ab initio* total-energy calculations using a plane-wave basis set. *Phys. Rev. B* **54**, 11169-11186 (1996).
28 Kresse, G. & Hafner, J. Ab initiomolecular dynamics for liquid metals. *Phys. Rev. B* **47**, 558-561 (1993).
29 Blöchl, P. E. Projector augmented-wave method. *Phys. Rev. B* **50**, 17953-17979 (1994).
30 Momma, K. & Izumi, F. VESTA 3 for three-dimensional visualization of crystal, volumetric and morphology data. *J. Appl. Crystallogr.* **44**, 1272-1276 (2011).
31 Wang, V., Xu, N., Liu, J.-C., Tang, G. & Geng, W.-T. VASPKIT: A user-friendly interface facilitating high-throughput computing and analysis using VASP code. *Comput. Phys. Commun.* **267**, 108033 (2021).
32 Hapala, P. *et al.* Mechanism of high-resolution STM/AFM imaging with functionalized tips. *Phys. Rev. B* **90**, 085421 (2014).
33 Nasibullin, R. T., Dimitrova, M., Valiev, R. R. & Sundholm, D. Orbital contributions to magnetically induced current densities using gauge-including atomic orbitals. *Chemical Science* (2025).
34 Liu, Z., Lu, T. & Chen, Q. An sp-hybridized all-carboatomic ring, cyclo[18]carbon: Bonding character, electron delocalization, and aromaticity. *Carbon* **165**, 468-475 (2020).
35 Krygowski, T. M. Crystallographic studies of inter- and intramolecular interactions reflected in aromatic character of π-electron systems. *J. Chem. Inf. Comput. Sci.* **33**, 70-78 (1993).
36 Poater, J., Duran, M., Solà, M. & Silvi, B. Theoretical evaluation of electron delocalization in aromatic molecules by means of atoms in molecules (AIM)

and electron localization function (ELF) topological approaches. *Chem. Rev.* **105**, 3911-3947 (2005).

37 Matito, E., Salvador, P., Duran, M. & Solà, M. Aromaticity measures from fuzzy-atom bond orders (FBO). The aromatic fluctuation (FLU) and the para-delocalization (PDI) indexes. *J. Phys. Chem. A.* **110**, 5108-5113 (2006).

38 Matito, E., Duran, M. & Solà, M. The aromatic fluctuation index (FLU): A new aromaticity index based on electron delocalization. *J. Chem. Phys.* **122**, 014109 (2004).

39 Stradi, D., Jelver, L., Smidstrup, S. & Stokbro, K. Method for determining optimal supercell representation of interfaces. *J. Phys.: Condens. Matter* **29**, 185901 (2017).

40 Jelver, L., Larsen, P. M., Stradi, D., Stokbro, K. & Jacobsen, K. W. Determination of low-strain interfaces via geometric matching. *Phys. Rev. B* **96**, 085306 (2017).

41 Baryshnikov, G. V., Valiev, R. R., Kuklin, A. V., Sundholm, D. & Ågren, H. Cyclo[18]carbon: Insight into electronic structure, aromaticity, and surface coupling. *J. Phys. Chem. Lett.* **10**, 6701-6705 (2019).
